# Regulatory capacity is not a scalar: metrics, perturbations, and viability in network physiology

**DOI:** 10.3389/fnetp.2026.1961712

**Published:** 2026-10-13

**Authors:** Lori Hogenkamp

**Affiliations:** Center for Adaptive Stress, Cincinnati, OH, United States

**Keywords:** allostasis, allostatic load, network physiology, neurodiversity, perturbation response, recovery burden, regulatory bandwidth, viability

## Abstract

Biological regulatory capacity is commonly represented by a scalar: a summed biomarker index, standardized composite, risk count, or single recovery parameter. Such measures can be useful, but they do not identify capacity independently of the conditions under which they are constructed. This article argues that regulatory capacity is not an intrinsic scalar property of an organism. It is a conditional relation among the architecture and current state of a regulatory system, the perturbations that reach it, the metric or cost functional used to evaluate its response, the viability criterion, and the time horizon. Near an operating state, systems with identical eigenvalue spectra can incur different integrated burdens and reverse their apparent ranking when the perturbation channel or burden metric changes. For a declared quadratic burden in a stable local linearization, the observability Gramian associated with a virtual cost output represents accumulated weighted recovery burden, and the measurement map, burden-evaluation map, and latent state representation must be distinguished. For network physiology, allostasis, and allostatic-load measurement, routine choices about biomarkers, scaling, thresholds, and challenge paradigms are therefore constitutive assumptions rather than neutral preprocessing. Biological adaptation is likewise not equated with stability, compensation, recovery, or continued viability: it is indexed to a specified level of organization, protected function, reference or counterfactual, time horizon, and outcome or cost criterion. Regulatory Bandwidth is developed as a conditional perturbation-response profile rather than a universal scalar. Its contribution is a measurement architecture that requires the regulatory system, perturbation ecology, burden criterion, viability endpoint, and horizon to be declared before scalarization. The primary falsifiable comparison is between a flexible scalar-capacity model, which predicts one preserved ordering across declared perturbation classes, and a conditional-profile model tested on held-out sessions, contexts, or perturbations. Rejection of the scalar model supports conditional structure without establishing architectural heterogeneity, which requires a further shared-architecture comparison; this distinction bears directly on neurodiversity and person-environment fit. A worked orthostatic-challenge specification shows how measured outputs, constructed burden, viability, measurement error, and mechanistic interpretation can be separated in practice. The framework preserves the usefulness of scalar summaries while restricting the claims they can support.

## 1 Introduction: the hidden scalar assumption

Stress physiology has long recognized that regulation is multisystem, dynamic, and context-dependent. Autonomic, neuroendocrine, immune, metabolic, and behavioral processes interact across timescales; the same exposure may be manageable in one state and destabilizing in another; and successful regulation may require anticipatory change rather than return to a fixed set point (McEwen, 1998; Sterling, 2012; Del Giudice et al., 2018). Network physiology makes this interdependence the object of analysis: organ systems are treated as dynamically interacting networks whose topology and coupling reorganize across states and timescales (Bashan et al., 2012; Bartsch et al., 2015; Ivanov, 2021). Yet when this complexity is measured, it is commonly compressed into a single number. Allostatic load is often operationalized as a count or composite of biomarkers placed in high-risk ranges. Other approaches sum standardized biomarker values, estimate latent factors, or construct algorithmic risk scores. These measures can predict morbidity, mortality, or functional decline, and their utility should not be dismissed (Seeman et al., 2001; Juster et al., 2010; Guidi et al., 2021). The problem is narrower and more fundamental: what, exactly, does the resulting scalar measure?

The answer is not supplied by the biomarkers alone. A composite depends on which variables were included, how each was scaled, whether high and low values were treated symmetrically, how systems were weighted, which thresholds defined risk, and whether the measurement captured a resting level, a response to challenge, or recovery afterward. The allostatic-load literature has documented substantial heterogeneity in biomarker selection and algorithm construction, including many different calculations applied to the same national dataset (Duong et al., 2017; Johnson et al., 2017; Carbone et al., 2022). These are usually treated as operationalization problems to be resolved through consensus. I argue that they reveal something deeper: there is no measure of regulatory capacity without a model of what departures matter, what demands are relevant, and what continued function the system is required to preserve.

Regulatory Bandwidth (RB) was introduced as the organism’s present coordinated regulatory capacity across interacting stress-responsive systems: the flexible range within which those systems can adjust to demand without functional destabilization (Hogenkamp, 2026). Section 4.1 formalizes functional continuity and functional destabilization relative to declared tolerances and a declared horizon; the term is used here in that sense. The originating paper deliberately left its final operational form open. That restraint matters. A static biomarker composite may index multisystem strain, but it does not by itself measure how a perturbation enters the system, how it propagates through coupled subsystems, how much biological or functional burden the resulting trajectory imposes, or whether the trajectory crosses a viability boundary. This paper formalizes that measurement problem.

The central claim is:

Regulatory capacity is not an intrinsic scalar. It is a relation among regulatory architecture and state, perturbation ecology, biological cost, and viability.

The mathematical tools used to support this claim are not new. Reactivity, transient growth, Lyapunov metrics, Gramians, input-output gains, and viability are established concepts in dynamical systems and control theory (Moore, 1981; Farrell and Ioannou, 1996; Neubert and Caswell, 1997; Trefethen and Embree, 2005; Aubin et al., 2011). The contribution proposed here is translational and theoretical: to show why those results change the interpretation of multisystem stress indices; to identify the biological assumptions currently hidden inside routine preprocessing choices; and to develop a testable, non-pathologizing account of why regulatory capacity may differ across people and environments without reducing either the person or the environment to a scalar cause.

## 2 What a scalar composite assumes

Suppose an index contains cortisol, heart-rate variability, C-reactive protein, glycated hemoglobin, and blood pressure. Before those quantities can be added, their different units must be removed or reconciled. Z-scoring accomplishes this statistically by expressing each observation in standard-deviation units relative to a reference sample. Quantile and clinical-cutoff methods accomplish it by converting values into risk indicators. Either move is defensible for a stated purpose. Neither is neutral.

Standardization establishes a geometry. In a simple unweighted sum of z-scores, a one-standard-deviation departure in cortisol is treated as commensurate with a one-standard-deviation departure in blood pressure or inflammation. The reference cohort determines the scale: changing the cohort can change the same person’s standardized profile without any change in that person’s physiology. A cutoff method makes a different commitment, treating departures on one side of a threshold as equivalent and those on the other side as categorically different. Weighting biomarkers by regression coefficients makes the outcome used to train the model part of the definition of load. Factor analysis makes covariance in a particular sample part of the definition. These choices may be appropriate, but each answers a different question.

The problem is not solved by adding more biomarkers. More variables can improve coverage while making the implicit metric harder to see. Nor is it solved automatically by data-driven weighting. A model optimized to predict mortality estimates risk under a particular population, follow-up period, and outcome definition; it does not thereby discover a context-free quantity called capacity. Prediction and construct identification are different achievements.

Allostatic load and Regulatory Bandwidth should not be collapsed. Allostatic load generally names cumulative multisystem burden or risk, whereas RB names present capacity under demand. Allostatic-load methods are relevant here because they are the most developed neighboring example of multisystem biomarker aggregation and because the original RB paper identified overlapping physiological domains. The argument is not that allostatic-load indices fail to measure a capacity they never promised to measure. It is that extending a resting multisystem composite into a measure of present regulatory capacity requires additional information about perturbation, trajectory, cost, and viability.

Three distinctions are therefore required.

First, state is not dynamics. A resting snapshot indicates where measured variables are at one time. It does not identify how the coupled system will respond to a perturbation.

Here and below, recovery denotes movement of measured states, outputs, or function toward a declared reference state or trajectory; it does not necessarily imply resolution of the underlying response process, restoration of every affected domain, or return to the original state (Section 4.1).

Second, return rate is not recovery burden. Two systems may return at similar asymptotic rates while traversing very different transient paths and incurring different energetic or functional costs.

Third, recovery burden is not viability—remaining within declared physiological or functional constraints over a declared horizon (Section 4.1). A trajectory can be costly yet remain viable, or cross a critical functional boundary despite a modest value under a chosen average-cost metric. Here and below, *collapse* is an umbrella term for crossing a declared functional or physiological boundary or leaving the modeled operating regime; its application-specific meaning is fixed in Section 4.1. It is a constraint or regime phenomenon, not merely a large norm.

These distinctions motivate the formal apparatus that follows.

## 3 A relational formulation of regulatory response

### 3.1 Architecture, state, and input

Let the regulatory dynamics of an individual or system i be written generally as

$${\dot{x}}_{i}=f_{G_{i}}\left(x_{i},u_{i},e,t;\theta_{i}\right)+\xi_{i}\left(t\right).$$

Here $x_{i}$ is a vector of physiological and functional states; $G_{i}$ denotes regulatory architecture, including network topology, coupling organization, and timescale structure; $\theta_{i}$ denotes parameters or calibration within that architecture; $u_{i}$ represents exogenous perturbations or imposed inputs; e represents environmental conditions; and $\xi_{i}$ represents unresolved fluctuations. This notation separates architecture from parameter values without assuming that either is directly observable from one response. Endogenous regulatory feedback is represented within the closed-loop dynamics and architecture; a formulation that models control action separately would assign distinct variables to disturbance and control.

Linearizing deviations about an operating equilibrium, the local response may be approximated as

$$\delta \dot{x}=J\delta x+B\delta u,$$

where J is the Jacobian of the dynamics at the current operating state and B specifies how a perturbation enters the modeled state dynamics. Both are necessary. The same internal dynamics can respond differently when a perturbation enters through a sensory channel, immune challenge, circadian phase shift, social demand, or metabolic constraint. A claim about capacity that specifies J but not the input channel is incomplete. Along a nonstationary nominal trajectory the corresponding matrices are generally time-varying; the constant J and B used below should therefore be understood as a local frozen-time approximation.

For readability, the deviation variable δ*x* is written as *x* from Section 3.2 onward. All subsequent quantities are deviations from the current operating state, and *y*_ref_ denotes the measured output at that state, so that *y* − *y*_ref_ = *Hx* in the noise-free local model.

*Perturbation* is the mathematically neutral term used in the formal model. A *challenge* is a bounded protocol used to elicit a response, a *demand* is an ongoing requirement for regulatory work, and a *stressor* is a perturbation or demand that threatens a declared function in interaction with the system’s architecture, state, and exposure history (Section 4.1). The terms are related but not interchangeable; a perturbation in the mathematical model need not constitute biological stress.

#### 3.1.1 State variables, measured outputs, and model status

The vector *x* has so far been described as physiological and functional states. Its status should be made explicit, because the results that follow are coordinate-dependent and because the distinction between what is modeled and what is measured carries most of the interpretive weight in this article. Write the system and its observation process separately:

$$\dot{x}=f\left(x,u\right)+\xi ,y=h\left(x\right)+\varepsilon ,\text{or locally }y=Hx+\varepsilon ,$$

where *x* is the chosen state representation, which may be latent; ξ is unresolved process variation within the dynamics, as introduced in Section 3.1; *y* is the vector of directly measured signals; *H* is the measurement map; and ε is measurement error arising in the observation process. The two noise terms are kept distinct: ξ perturbs the trajectory, ε corrupts the record of it, and only ε contributes the bias term derived in Section 9.1. The architecture *G*, calibration θ, and Jacobian *J* are likewise model quantities, none of them assumed directly visible in a biological data stream.

Nothing in the argument requires *x* to be a hypothesized mechanistic state. But neither may an arbitrary set of measured signals be substituted for one. A vector of observations is not in general a dynamically sufficient state: its future evolution may depend on variables that were not measured and on exposure history, in which case its trajectory is not determined by its present value. Where the measured variables do constitute a dynamically sufficient representation over the studied horizon, one may take *y* = *x*, or *H* = *I* in the local linear form. Otherwise the observation process must remain explicit, or the observed series must be augmented—with lags, or with inferred latent variables—before it is treated as a state.

The burden evaluation is a separate declaration again. Let *z* = *L* (*y* − *y*_ref_) denote the burden-relevant output, where *L* states which observed departures count and how strongly. In the noise-free local model this gives *z* = *C*_y_ *x* with *C*_y_ = *LH*. The two maps answer different questions. *H* specifies what can be observed. *L* specifies what is to count as burden for the biological question at hand. Neither is supplied by the organism, and conflating them is the specific error this article is concerned with: a composite index inherits commitments from both and reports a single number as though it had recovered a property.

The system–environment partition is an analytic choice on the same footing: a social demand may be modeled as an input through *B* or as part of the coupled dynamics in *G*, and the two choices attribute burden differently to the same events.

Declaration is necessary but not sufficient. Stating a state space, a measurement map, and an evaluation map makes a claim inspectable; it does not make it biologically valid. The chosen representation must also support identification from available data, stable out-of-sample prediction, and transport across the perturbations and contexts to which the resulting capacity claim is intended to apply. Those are the requirements Section 8 and Section 9 operationalize. Where a state space is learned from data rather than specified in advance, the same requirements apply to the procedure that produced it (Section 6.5).

### 3.2 Stability, transient growth, and the metric problem

If all eigenvalues of J have negative real parts, the linearized system is asymptotically stable: sufficiently small perturbations eventually decay. One scalar summary of the long-time behavior is the asymptotic relaxation rate

$$\alpha \left(J\right)=-\max_{k}Re\lambda_{k}\left(J\right)>0.$$

The rate α constrains long-time recovery but does not determine the full transient. In non-normal systems, non-orthogonal modes can combine to produce transient growth before asymptotic recovery, so eigenvalues alone may underestimate finite-time excursions (Farrell and Ioannou, 1996; Neubert and Caswell, 1997; Trefethen and Embree, 2005). Directed and hierarchical networks commonly display non-normal structure, making the issue relevant to network physiology (Asllani et al., 2018).

Transient growth is not a coordinate-free biological fact; it is growth relative to a norm. For a fixed positive-definite metric *W*, the metric-specific reactivity is

$$\nu_{W}\left(J\right)=\lambda_{\max}\left[W^{-1/2}\frac{WJ+J^{T}W}{2}W^{-1/2}\right].$$

Positive *ν*_*W*_ *(J)* means that some initial directions grow instantaneously in the *W*-norm. If *ν*_*W*_ *(J) ≤ 0* the *W*-norm is non-increasing; if *ν*_*W*_ *(J) < 0* it contracts strictly. For any Hurwitz matrix *J* and any positive-definite matrix *Q*, the Lyapunov equation

$$J^{T}P+PJ=-Q$$

has a unique positive-definite solution *P* (Khalil, 2002). In the quadratic measure *V(x) = x^T^Px*, *dV/dt = −x^T^Qx < 0* for all *x ≠ 0*, and therefore *ν*_*P*_ *(J) < 0*. Thus a stable linear system that appears transiently amplifying in one metric can be monotone in another. Non-normality and reactivity remain useful descriptions once a physically meaningful metric is fixed; they cannot, by themselves, supply that metric.

This is not a reason to abandon transient analysis. It identifies the missing biological question: what notion of distance or burden should physiology preserve under a change of coordinates? In fluid dynamics, the metric may be kinetic energy. In an electrical system, it may be stored or dissipated energy. In organism-level stress physiology, the relevant metric cannot be assumed to be Euclidean distance among standardized biomarkers. It must be justified by a biological or functional cost.

### 3.3 A fixed cost functional and the observability Gramian

For illustration, suppose the locally meaningful burden of a deviation is quadratic:

$$q\left(x\right)=x^{T}Wx,\;W≽0,$$

where W encodes which departures matter and how strongly. For the autonomous local system $\dot{x}=Jx$ , define integrated recovery burden as

$$C_{W}\left(x_{0}\right)=\int_{0}^{\infty }x{\left(t\right)}^{T}Wx\left(t\right)\;dt.$$

When *W* is singular, *q* is an output cost rather than a norm; observability determines whether the integrated Gramian is nevertheless positive definite.

When J is stable,

$$C_{W}\left(x_{0}\right)=x_{0}^{T}G_{W}x_{0},$$

where $G_{W}$ is the observability Gramian satisfying

$$J^{T}G_{W}+G_{W}J=-W.$$

Three distinct conclusions should be kept separate. First, a unique solution *G*_*W*_ to the algebraic Lyapunov equation exists whenever *J* is Hurwitz. Second, that Lyapunov solution is an observability Gramian when the right-hand side has the form *W = C* ^*T*^ *C*, which is available for any *W ⪰ 0*; *G*_*W*_ is then the observability Gramian of the pair *(J, C)*. Third, *G*_*W*_ is positive definite if and only if *(J, C)* is observable, and is positive semidefinite otherwise. Any later step that treats *G*_*W*_ as positive definite or invertible therefore carries an observability assumption (Moore, 1981).

This is standard control theory, not a new definition or theorem. Its value here is diagnostic. The dynamics J do not determine burden alone. Burden depends jointly on J , the initial perturbation $x_{0}$ , and W . Under a coordinate transformation $\tilde{x}=Sx$ , the value remains invariant only when the metric transforms covariantly, $\tilde{W}=S^{-T}WS^{-1}$ . Rescaling the state variables while leaving the implicit metric unchanged changes the reported burden. Routine standardization therefore makes a substantive choice unless its relationship to biological cost is defended.

The quadratic assumption is only a tractable starting point. Physiological cost may depend on flux, rate of change, saturation, duration above threshold, path history, or interactions with reserve. The point does not depend on quadratic cost being correct. On the contrary, the likelihood that it is incomplete strengthens the requirement to make the cost functional explicit.

#### 3.3.1 What the Gramian represents here

Because the observability Gramian appears in more than one role in the control-theory literature, the role in use here should be stated. Two observability questions arise and should not be conflated. The first concerns reconstruction—whether the relevant latent state directions can be identified from the instruments, a property of (*J*, *H*)—and is a precondition for the empirical program of Section 9, but not what the Gramian evaluates. The second concerns evaluation—whether any nonzero initial departure can produce identically zero declared cost output along the subsequent trajectory, a property of (*J*, *C*)—and is the question that bears on *G*_W_.

Observability of (*J*, *H*) is a structural property of the idealized local model; practical recoverability from finite data depends additionally on sampling, excitation, measurement error, model adequacy, and parameter identifiability. Neither property implies controllability or ease of regulation: that a direction is well expressed in a measurement, or well priced by a cost, says nothing about whether it can be steered, which requires a controllability analysis, an actuator structure, and a controller. The inference from “well observed” to “readily regulated by feedback” does not hold, and nothing here depends on it.

For a declared burden weight *W* and stable *J*, *G*_W_ is the unique solution of $J^{T}G_{W}+G_{W}\;J=-W$ , and 𝒞_W_(*x*_0_) = $x_{0}^{T}G_{W}\;x_{0}$ is the accumulated weighted burden of the trajectory beginning at *x*_0_. It is the observability Gramian associated with the virtual cost output *z* = *Cx*. A larger value denotes greater burden under the declared conditions—not greater capacity, and not greater estimator precision.

Two routes to a cost output should be distinguished. A burden may be declared directly on the model state through *W*_x_ ⪰ 0, with *C*_W_ denoting any factor satisfying $W_{x}=C_{W}^{T}C_{W}$ . Alternatively, a burden may be constructed from measured outputs through *z* = *L* (*y* − *y*_ref_), which locally induces *C*_y_ = *LH* and $W_{y}=H^{T}L^{T}LH$ . The two routes coincide only when the declared state-space burden is the one induced by the measurement and evaluation maps, that is when *W*_x_ = *W*_y_. In that case *C*_y_ is one admissible factor of the same *W*, and nothing depends on which factor is selected: *G*_W_ solves a Lyapunov equation in *W* alone, and

$${\left\|Ce^{Jt}x_{0}\right\|}^{2}={\left(e^{Jt}x_{0}\right)}^{T}W\left(e^{Jt}x_{0}\right)$$

depends only on *W*. If *W*_x_ ≠ *W*_y_, the two constructions represent different burden questions rather than different factorizations of one question; a study that declares one while computing the other has changed its question without saying so.

*G*_W_ ≻ 0 if and only if $Ce^{Jt}x_{0}≢0$ for every *x*_0_ ≠ 0: no nonzero initial departure produces identically zero cost output over the trajectory. This is a condition on the whole trajectory, not on instantaneous weighting. A direction ignored at *t* = 0 may later feed a priced output through the dynamics; conversely, an unobservable invariant subspace remains cost-invisible indefinitely.

Once the measurement map is explicit, *W_y_ =H^T^L^T^LH* has rank at most min (rank *L*, rank *H*), so *W*_y_ is singular whenever fewer independent channels are measured than the model carries states—the usual empirical situation. The quadratic form is then an output cost rather than a norm on the state space, and singularity does not by itself settle whether the burden is degenerate. Take the two architectures of Figure 1, measure the autonomic and inflammatory channels but not the neuroendocrine one, and weight the measured channels equally: the induced *W*_y_ is identical for both systems and has rank two of three. In System A, where the unmeasured neuroendocrine state drives nothing, a unit departure in it accrues exactly zero burden and *G*_W_ is singular; in System B, where the same state drives the measured inflammatory channel, the identical departure accrues substantial burden and *G*_W_ is positive definite. The adequacy of a measurement set therefore cannot be judged from the channel list alone; it depends on how unmeasured states propagate into measured, priced ones, and so on the coupling structure a study is often trying to establish.

**FIGURE 1:**
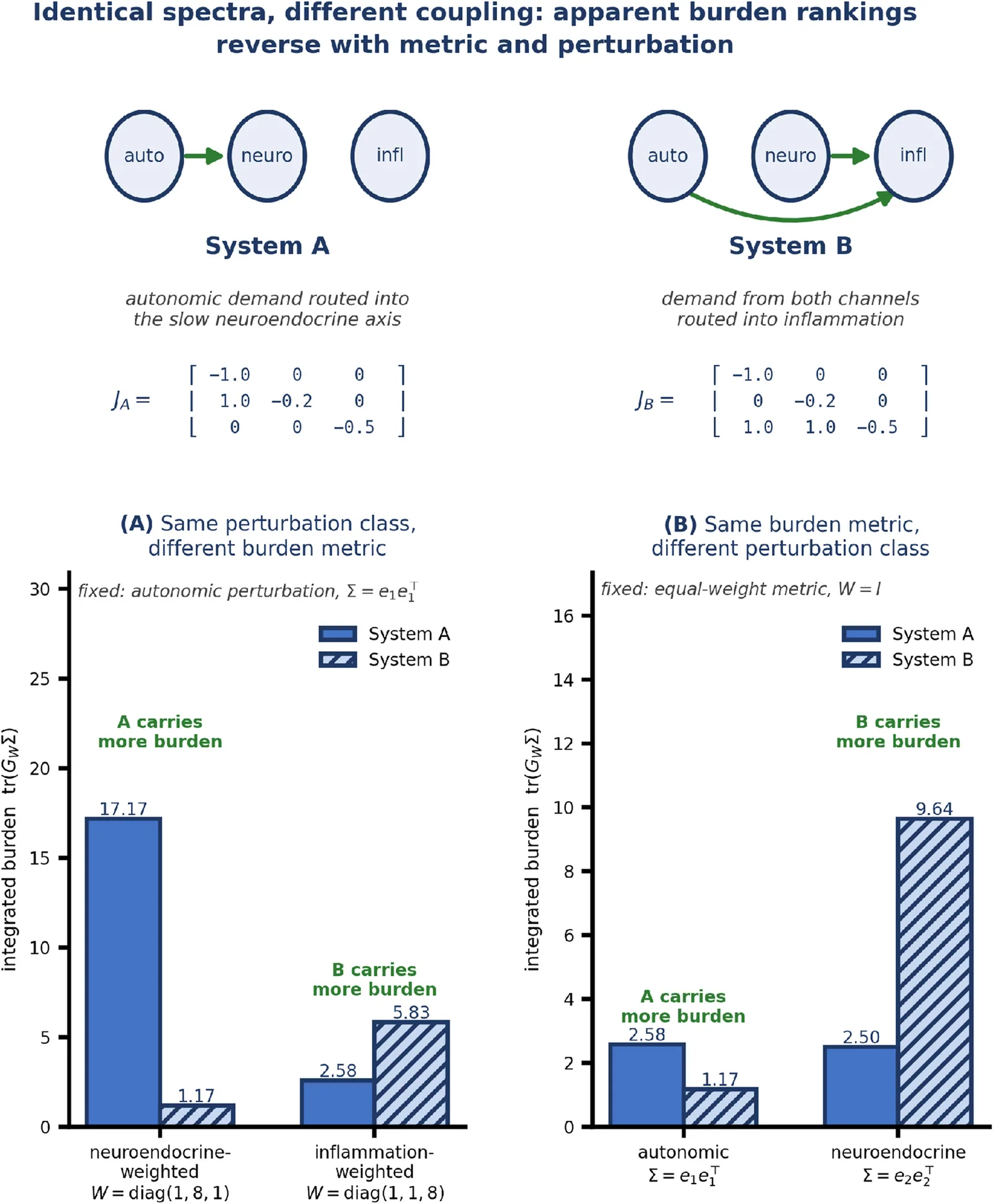
Identical spectra, different coupling: apparent burden rankings reverse with metric and perturbation. Systems A and B are illustrative stable local linear networks over three states—autonomic (x_1_), neuroendocrine (x_2_), and inflammatory (x_3_)—with self-decay rates −1.0, −0.2, and −0.5 respectively. The two Jacobians share the eigenvalue spectrum {−1.0, −0.5, −0.2} and therefore the same asymptotic relaxation rate α = 0.2; they differ only in directed off-diagonal coupling, and every nonzero coupling has magnitude one, so the reversals shown are produced by coupling topology alone. Self-decay terms are omitted from the network schematics. Burden is the integrated quadratic cost $C_{W}\left(x_{0}\right)=\int_{0}^{\infty }x\left(t\right){\;}^{T}Wx\left(t\right)\;dt=tr\left(G_{W}\;\Sigma \right)$ , evaluated for a unit initial displacement on a single channel; $\Sigma =x_{0}x_{0}^{T}$ is therefore rank one and $\text{tr}\left(G_{W}\;\Sigma \right)=x_{0}^{T}G_{W}\;x_{0}$ , so the two expressions coincide for the values plotted. **(A)** The perturbation class is held fixed (autonomic, $\Sigma =e_{1}e_{1}^{T}$ ) while the burden metric changes: System A incurs 14.7 times the burden of System B under a neuroendocrine-weighted metric (W = diag (1, 8, 1)), whereas System B incurs 2.3 times the burden of System A under an inflammation-weighted metric (W = diag (1, 1, 8)). **(B)** The burden metric is held fixed (equal weight, W = I) while the perturbation class changes: System A incurs 2.2 times the burden of System B under an autonomic perturbation, whereas System B incurs 3.9 times the burden of System A under a neuroendocrine perturbation. Neither system therefore carries a context-free capacity rank. Lower integrated burden corresponds to greater apparent capacity only relative to the declared perturbation class, evaluation metric, and time horizon; it does not establish an ordering of the two systems as such. Values are computed from the illustrative local linear models shown and are not empirical observations.

The infinite-horizon Gramian above is a limiting case of

$$G_{W}\left(T\right)=\int_{0}^{T}e^{J^{T}t}We^{Jt}\;dt,C_{W,T}\left(x_{0}\right)=x_{0}^{T}G_{W}\left(T\right)x_{0},$$

with *G*_W_(*T*) → *G*_W_ as *T* → ∞ for Hurwitz *J*. Empirical challenge studies require a finite, prospectively declared *T*, which is why the horizon is carried as an explicit argument of the relation. In the example of Figure 1 the burden orderings are present at the shortest horizon examined and retained at every horizon tested thereafter, and by roughly two time constants of the slowest mode every finite-horizon burden is within a few percent of its limit; the reversals are properties of the transient trajectory, not artifacts of an unbounded window. A horizon short relative to the slowest mode that *W* prices recovers the ordering while understating the burden, so *T* must be declared relative to the modes the metric weights—subject to the countervailing constraint of Section 9.1 that lengthening *T* also accumulates measurement error.

### 3.4 Perturbation ecology

Worst-case response is rarely the ecological question. Organisms encounter distributions of perturbations. If initial perturbations are zero-mean with covariance Σ , then under the quadratic local formulation,

$$E\left[C_{W}\right]=tr\left(G_{W}\Sigma \right).$$

For perturbations with non-zero mean *μ* the expected burden acquires an additional term *μ^T^G*_*W*_ *μ*; equivalently, the expression above holds exactly when *Σ* is taken as the uncentered second-moment matrix *E[x*_*0*_ *x*_*0*_ *^T^]*. The zero-mean case is used throughout.

If *W = C^T^C* and *Σ = RR^T^*, the expected cost can be written as

$$tr\left(G_{W}\Sigma \right)={∥C{\left(sI-J\right)}^{-1}R∥}_{H_{2}}^{2}$$

under an impulse or random-initial-condition interpretation. Here R factors the initial-condition covariance; it is neither the measurement map H, nor the evaluation map L, nor the physical input matrix B. Because *Σ* is not a covariance of persistent forcing, I use “H_2_-type” only by analogy—that is, the quantity has the same algebraic form as an H_2_ norm without arising from the same forcing assumptions. The expression makes the relational structure explicit. *G*_*W*_ describes how the network converts a specified initial departure into accumulated burden under the chosen metric; *Σ* captures the second-moment geometry of those departures—their directions, amplitudes, and covariance. *Σ* does not encode event rate, switching frequency, temporal ordering, duration, or spectral content. Those properties require a driven formulation with an explicit input matrix *B* and a temporally specified input process, so environments with the same marginal covariance can differ substantially in perturbation ecology. Neither factor alone is regulatory capacity in the ecological sense.

This provides a formal route to person-environment fit. Two architectures can have similar average burden under one initial-departure distribution or driven input process and sharply different burden under another. A highly predictable, low-switching environment and a noisy, rapidly switching environment differ primarily in temporal input structure, even when average demand or marginal covariance is similar. Accommodation—an external change in input channel, intensity, timing, predictability, environmental structure, or distribution of regulatory work (Section 4.1)—may change the encountered perturbation distribution, input channel, temporal input process, or operating state without changing the person’s architecture. Its effect can nevertheless be biologically large.

## 4 Viability: why burden is not enough

Integrated burden remains a cost-based summary. Regulation also involves constraints, the domain addressed by viability theory (Aubin et al., 2011). Let V *(t)* denote the time-dependent region of states compatible with continued physiological and functional viability for the process under study. A perturbation is viable over a horizon T when its trajectory remains in that region throughout:

$$x_{u}\left(t\right)\in V\left(t\right)\;for\;all\;t\in \left[0,T\right].$$

For a specified class of admissible initial displacements or input signals U , define the viable perturbation set

$$A_{T}=\left\{u\in U:x_{u}\left(t\right)\in V\left(t\right)\;\forall t\in \left[0,T\right]\right\}.$$

This object is set-valued and directional. A system may tolerate large perturbations along one channel and very little along another. The geometry or measure of $A_{T}$ depends on the architecture, current state, environment, time horizon, and viability criterion. A scalar summary can be computed from it—for example, the probability that a perturbation drawn from ecology π remains viable—but such a scalar is conditional on π and V :

$$R_{T}={\mathrm{Pr}}_{u∼\pi }\left(u\in A_{T}\right).$$

This is the sense in which Regulatory Bandwidth is better treated as a relation or profile than as an intrinsic amount. A useful operational representation would retain at least:

1. The viable perturbation set or probability under a stated perturbation ecology;
2. The distribution of recovery burdens $\Phi \left[u\right]$ among viable responses;
3. Recovery time and residual displacement; and
4. The channels along which viability is most easily lost.

The formulation also separates two mathematical questions that have been repeatedly conflated. Local input-output analysis asks how a system is displaced and what the excursion costs. Global nonlinear analysis asks whether the system leaves the domain in which a local model is valid or enters a different persistent regime. Gramians do not characterize such global transitions, and global state-space geometry does not determine a biologically meaningful recovery metric. The present local-burden analysis and separate global nonlinear analyses are therefore complementary, not interchangeable.

### 4.1 Viability is not adaptation

The viability formulation above establishes whether a trajectory remains within declared physiological or functional constraints over a specified horizon. That condition is not equivalent to biological adaptation. Viability is a property of a trajectory relative to a constraint set; adaptation is an indexed biological judgment about the consequences of a response.

In this article, a response is designated adaptive only relative to a specified level of biological organization, target function, reference state or counterfactual, time horizon, and outcome or cost criterion. Adaptation may involve resistance, compensation that preserves function, recovery toward a prior trajectory, or reorganization into a different viable state; it does not require return to the original state, and it does not imply optimality, absence of cost, or benefit at every biological level. Conversely, continued viability does not establish adaptation. A response may preserve short-term function through costly compensation while producing delayed burden, constraining another function, or narrowing the range of perturbations that can be managed later, and may therefore be adaptive for one function or horizon and maladaptively stabilized at another.

In this article, *collapse* is an umbrella term rather than a single biological event. Each application must declare whether it refers to crossing a functional or physiological viability boundary, departure from the local operating regime, transition to another attractor, clinical decompensation, or organismal loss of viability. These meanings should not be treated as interchangeable. Table 1 fixes the terminology used in the remainder of the article.

**TABLE 1:** Operational distinctions among perturbation, viability, recovery, and adaptation.

Term cluster	Operational use in this article	Required distinction
Perturbation, challenge, demand, stressor	A *perturbation* is a mathematically neutral input or initial displacement. A *challenge* is a bounded experimental or naturally occurring perturbation used to elicit a measurable response. A *demand* is an ongoing requirement for regulatory work. A *stressor* is a perturbation or demand that, in interaction with the system’s architecture, state, and history, materially increases regulatory work or threatens a specified function	Not every perturbation is a biological stressor. Stressor status is relational rather than an intrinsic property of an event, and depends on intensity, timing, predictability, channel, state, and person–environment fit
Stability	A dynamical property of an operating state or regime: Whether nearby departures decay, persist, or grow. Local asymptotic stability means that sufficiently small departures eventually decay	Stability does not establish low burden, adaptation, resolution, or long-term viability. A costly compensatory regime or a maladaptively stabilized state may itself be dynamically stable
Viability, functional continuity, functional destabilization, collapse	*Viability* means remaining within predeclared physiological or functional constraints over horizon *T*. *Functional continuity* is preservation of a specified target function within declared tolerances; *functional destabilization* is failure to maintain that threshold. *Collapse* denotes crossing the boundary or leaving the regime specified for the application	Continued viability does not establish adaptation or health. Functional destabilization does not necessarily imply global failure, and a threshold crossing does not by itself establish an attractor transition
Compensation and accommodation	*Compensation* is internal recruitment, redistribution, or intensification of regulatory processes that preserves a target function despite disturbance. *Accommodation* is an external change in input channel, intensity, timing, predictability, environmental structure, or distribution of regulatory work that changes the perturbation ecology	Preserved outward performance may conceal substantial compensatory burden. Accommodation need not change the person’s architecture or normalize behavior; it may improve viability by reducing the cost of maintaining function
Recovery and resolution	*Recovery* is movement of measured states, outputs, or functional performance toward a declared reference state or trajectory. It may be partial, multidomain, or directed toward a new viable regime. *Resolution* is termination of the response-generating process under study, including the absence of functionally consequential continuing activation or residual reprogramming over the declared horizon	Return of selected measurements toward baseline does not establish resolution. Recovery need not restore the original state, repay accumulated cost, restore every affected domain, or recover future capacity. Resolution may culminate in a different viable organization rather than a return to the prior baseline
Adaptation and maladaptive stabilization	*Adaptation* is an indexed judgment that, relative to an explicit comparator, a response improves preservation, restoration, or sustainable reorganization of a specified function at a specified biological level and over a specified horizon. *Maladaptive stabilization* is a persistent or self-maintaining regulatory organization that may preserve immediate function or originate as a protective response but subsequently increases burden, impairs another function, or narrows future viable response under the declared criterion	Adaptation is not synonymous with change, persistence, compensation, recovery, resilience, or viability. “Maladaptive” is not an intrinsic defect or moral judgment; it is relational to the level, function, environment, horizon, comparator, and consequences being evaluated
Biological memory and regulatory reprogramming	Prior exposure may alter current state, calibration, input sensitivity, viability boundaries, and—under a stronger architectural hypothesis—coupling organization	Responses to repeated perturbations cannot be assumed to be exchangeable. A changed response may reflect state or calibration and does not by itself establish architectural change
Chronicity	Persistence of a response, state, or regulatory organization over time. Chronicity may arise from continued exposure, unresolved activation of an initially protective response, or qualitative reprogramming into a self-maintaining regime	Duration alone does not distinguish prolonged activation from regulatory reprogramming. Two responses of equal duration may therefore represent different biological processes and carry different implications for future viability

Adaptation, viability, recovery, resolution, and collapse are indexed terms. Their use requires specification of the biological level, target function, reference state or counterfactual, time horizon, and outcome or cost criterion. These definitions establish the usage adopted in this article; they do not imply that neighboring literatures use each term identically.

Regulatory Bandwidth accordingly characterizes conditional perturbation tolerance, accumulated burden, recovery dynamics, residual state, and continued viability under declared conditions; it does not independently determine whether a response is biologically adaptive.

History dependence (Table 1) may appear as habituation, sensitization, tolerance, priming, learned prediction, or persistent molecular or systems-level reorganization. These manifestations differ in mechanism and timescale and should not be collapsed into a single category of incomplete recovery. Prior exposure must therefore remain part of the formulation. In principle, a dynamically sufficient state would contain all history relevant to future evolution. Empirical state vectors rarely meet that standard. Exposure history ℋ_t_ may therefore condition the current state, calibration, and viability region:

$$x_{t}=x\left(H_{t}\right),\theta_{t}=\theta \left(H_{t}\right),V_{t}=V\left(H_{t}\right).$$

History acting through *x*_t_ and θ_t_ is the ordinary case and can represent the manifestations listed above. Over developmental or plastic timescales, exposure history may also alter *G*_t_, but that possibility is not on the same evidential footing:

$$G_{t}=G\left(H_{t}\right)$$

is an architectural hypothesis: a claim that history has changed the coupling topology, timescale organization, or input–output structure must meet the evidence standard of Section 7 and cannot be inferred from a changed response alone. Chronicity must consequently be evaluated by both duration and regulatory organization: persistence may represent continuing activation of the original response, an unresolved demand, or transition to a qualitatively different and self-maintaining regime. An analogous distinction has been drawn at the cellular scale for the integrated stress response, where chronicity may reflect qualitative regulatory reprogramming rather than merely longer activation of the acute response (Altintas et al., 2026). The example is used here to illustrate the recurrence of the duration–reprogramming distinction across levels of organization, not to imply that organism-level Regulatory Bandwidth reduces to the integrated stress response pathway.

## 5 Regulatory Bandwidth as a conditional profile

The published RB construct names present coordinated capacity, not a material resource or substance that is literally spent (Hogenkamp, 2026). The present analysis sharpens that definition by specifying its arguments. In compressed notation:

$$RB=RB\left(G,\theta ,x_{t};B,\pi ;\Phi ,V,T\right).$$

The semicolons are conceptual, not algebraic. They separate three domains:

- System: architecture G , calibration θ , and current operating state $x_{t}$ ;
- Ecology: input channels B and perturbation distribution π ; and
- Evaluation: burden functional Φ (the quadratic $C_{W}$ of Section 3.3 is one instance), viability constraint V , and time horizon T .

No universal ordering of systems follows without fixing the latter two domains. System A may show broader viable response to sensory detail and narrower response to rapid social ambiguity, while system B displays the reverse. Calling one globally higher-bandwidth discards the architecture-specific robustness-fragility profile that the measurement should reveal.

This conditionality does not make the construct unfalsifiable. It makes the conditions of comparison explicit. Once the perturbation class, evaluation metric, and viability endpoint are fixed, responses can be compared, predictions can fail, and models can be rejected. What is not justified is moving silently from a conditional measurement to an unconditional ranking of persons.

### 5.1 A worked example: rank reversal with identical spectra

The conditionality claimed above has arithmetic consequences that can be exhibited in a system small enough to check by hand. Consider two three-dimensional local models over an autonomic state *x*_1_, a neuroendocrine state *x*_2_, and an inflammatory state *x*_3_, with self-decay rates −1.0, −0.2, and −0.5. Let System A route autonomic demand into the slow neuroendocrine axis, and let System B route demand from both the autonomic and neuroendocrine channels into inflammation (Figure 1). Every nonzero off-diagonal coupling has magnitude one.

The example declares burden directly on the model state—*W*_x_ in the terminology of Section 3.3.1—and uses deviation coordinates in which all three states are treated as fully and exactly measured: *y* = *x*, *H* = *I*, *y*_ref_ = 0, and ε = 0. Choosing an observed-space evaluation factor *L* satisfying $L^{T}L=W_{x}$ gives $Wy=H^{T}L^{T}LH=W_{x}$ . The direct state-space and measurement-induced burden constructions therefore coincide by design, and the reversal cannot be attributed to measurement error, incomplete observation, or incompatibility between the two burden definitions. That guarantee is withdrawn in Section 9.1, where the measured outputs need not constitute the full state and compatibility must be examined rather than assumed.

Both Jacobians are lower triangular with the same diagonal. They therefore share the eigenvalue spectrum {−1.0, −0.5, −0.2}, the same asymptotic relaxation rate α = 0.2, and the same equilibrium in the deviation coordinates. No eigenvalue-based summary distinguishes them, and a resting composite constructed from the declared equilibrium-state variables assigns them the same value.

Their integrated burdens do not coincide. Holding the perturbation fixed as a unit autonomic displacement and changing only the cost functional, System A carries 14.7 times System B’s burden under a metric that weights neuroendocrine excursion, while System B carries 2.3 times System A’s burden under a metric that weights inflammatory excursion (Figure 1A). Holding the cost functional fixed at equal weights and changing only the perturbation channel, System A carries the larger burden under an autonomic perturbation and System B carries the larger burden under a neuroendocrine perturbation (Figure 1B). The ordering of the two systems reverses under a change of metric with the perturbation fixed, and again under a change of perturbation with the metric fixed.

Three points follow. First, the reversal does not depend on fine-tuning: every nonzero coupling has magnitude one and the spectra are identical, and the coupling patterns were deliberately selected as a transparent existence proof. The example establishes that topology alone can produce such reversals under minimal conditions, not how common they are in biological systems. Second, the reversal is not measurement error or biological noise; it is a deterministic property of the Gramian and would recur exactly in any replication of the calculation. Third, and consequently, the question “which of these two systems has greater regulatory capacity?” has no answer until the perturbation class and cost functional are declared. Once those are fixed, the ordering is retained at every horizon examined, but the burden values vary with *T*, so comparisons of magnitude additionally require a declared horizon. The scalar habit does not merely lose precision; it poses a question the mathematics does not answer.

The example is a formal demonstration on a local linear model, not a biological observation. Its role is to establish that the conditionality is real and consequential before Section 9 sets out how it would be tested on measured trajectories.

### 5.2 Relationship to neighboring constructs

Regulatory Bandwidth overlaps substantially with established accounts of allostasis, resilience, reserve, robustness, and adaptive capacity; its incremental contribution is methodological, requiring present capacity to be represented as a conditional relation among the system, the perturbations that reach it, and the criteria by which burden and continued function are judged. Table 2 identifies the convergence and the additional commitments.

**TABLE 2:** Regulatory Bandwidth in relation to neighboring constructs.

Construct	What the established construct captures	Convergence with Regulatory Bandwidth	Incremental contribution of Regulatory Bandwidth in this article
Allostasis	Stability achieved through anticipatory and context-sensitive regulatory change rather than maintenance of fixed set points	Both treat regulation as dynamic, predictive, multisystem, and dependent on environmental demand	Allostasis describes the regulatory process. RB specifies the conditional response capacity presently expressed by that process: Which perturbations remain viable, what they cost, how recovery unfolds, and where fragility is concentrated under declared conditions. RB does not replace allostasis
Allostatic load	Cumulative multisystem burden, biological risk, or physiological strain associated with repeated or sustained regulatory demand	Both concern interacting stress-responsive systems and the biological consequences of regulatory work	Allostatic load is primarily history-facing; RB is response-facing and present-state dependent. The same resting load or biomarker composite may coexist with different perturbation-response trajectories, recovery burdens, and viability profiles. The claim is not that allostatic-load indices fail to measure a capacity they were not designed to measure
Physiological or dynamic resilience	Resistance to displacement, magnitude of response, recovery rate, recovery completeness, or reorganization following challenge	This is RB’s closest neighboring construct. Both require challenge-response measurement and may be multidimensional and context-dependent	RB makes the factorization among regulatory system, perturbation ecology, and evaluation criteria constitutive of the measurement. It requires perturbation channel, burden metric, viability criterion, and time horizon to be declared and retains the conditional response profile before scalarization. RB does not claim that challenge-response measurement or multidimensional resilience is new
Physiological reserve	The margin between ordinary functioning and maximal or failure-level performance, often estimated within a particular organ or functional domain	Both concern the room available before function becomes compromised	RB treats available capacity as coupled, directional, and perturbation-specific, rather than as a general stock. Two systems with similar estimated reserve may route the same demand differently and therefore incur different burdens or lose different functions first. Reserve may contribute to an RB profile without being identical to it
Robustness	Maintenance of a specified function despite a specified class of disturbances, including robustness–fragility trade-offs	Both reject context-free claims of strength and recognize that robustness in one direction may coexist with fragility in another	RB adds the distinction among preserved performance, accumulated burden, recovery, residual state, and viability. It also incorporates the encountered perturbation ecology: Which fragility directions are actually engaged, and how often. A fully specified robustness analysis may represent much of the same structure; RB assembles it specifically as a measurement architecture for present multisystem regulatory capacity
Adaptive capacity	The broad ability of a system to adjust, reorganize, or maintain function under changing conditions	Both are relational and context-sensitive and do not require return to an original state	RB provides a more constrained operational form. It identifies the declarations required before adaptive capacity can be measured and compared, and it yields a direct empirical test between a scalar-capacity model and a conditional-profile model. RB is not proposed as a replacement for the broader adaptive-capacity concept
Context-dependent stress-response and susceptibility models	Variation in outcome as a function of exposure, individual susceptibility, resources, and environmental context	Both predict person-by-environment interactions and reject the assumption that the same exposure has the same meaning for every system	RB formalizes the interaction in terms of input channels, coupling organization, trajectory burden, viability, and time horizon. It predicts that accommodations will have channel-specific effects when they reduce demands concentrated along a system’s fragility directions, rather than producing only a general main effect of support

No component of Regulatory Bandwidth is claimed to be absent from all neighboring frameworks; the proposed contribution is to require their conjunction in the operationalization and interpretation of present regulatory capacity.

The closest comparison is with dynamic resilience. A sufficiently rich resilience framework can represent resistance, recovery, reorganization, channel specificity, and person-by-challenge interactions. The novelty claimed here is therefore not the discovery that biological capacity is dynamic, multidimensional, or context-dependent, and RB is not proposed as a replacement for these constructs. The distinction is that RB treats the conditional structure as the primary measurement object rather than as an optional qualification of an underlying person-level score. A purpose-specific scalar may subsequently be computed, but its interpretation remains restricted to the declared perturbation ecology, metric, viability criterion, and horizon.

Figure 1B exhibits the formal structure of the observation that anchors this distinction: two systems at the same modeled equilibrium in the declared deviation coordinates, with the same asymptotic relaxation rate, reverse their burden ordering when only the perturbation channel changes, so a resting composite built from those equilibrium-state variables assigns them the same value. What the formal example cannot provide is the empirical anchor: it does not define physiological reserve or show that the crossover occurs in measured systems. The discriminating observation would be the same crossover among measured systems additionally matched on a conventional reserve estimate, replicating across sessions and predicting response to a held-out perturbation or a channel-matched accommodation. A dynamic-resilience framework can represent that observation by retaining challenge-specific response structure; under the present account, that conditional structure is the measurement object called Regulatory Bandwidth. The held-out comparison of Section 8.1 supplies the test.

## 6 Consequences for allostatic-load and multisystem-stress measurement

### 6.1 Composite indices remain useful—but answer a narrower question

A biomarker composite can summarize deviation or risk under a declared algorithm. It may predict future outcomes and provide a practical epidemiological indicator. The present argument does not require abandoning such tools. It requires naming them accurately. A resting composite is an index of selected biomarker states relative to a specified reference and weighting scheme. Without perturbation and recovery data, it is not a direct measure of regulatory response geometry.

This distinction may explain why different allostatic-load algorithms can all show predictive validity while disagreeing about individuals. They instantiate different metrics, reference distributions, thresholds, and outcomes. Agreement is an empirical property to be demonstrated, not guaranteed by their sharing a conceptual label.

### 6.2 Standardization should be treated as model specification

Studies should report how conclusions change under plausible scaling and weighting schemes, much as robustness analyses examine sensitivity to model assumptions. At minimum, investigators should state:

- Why each state variable belongs in the regulatory model;
- What reference population determines its scale;
- Whether the metric is intended to represent energetic, physiological, functional, or prognostic burden;
- How signs, nonlinear thresholds, and protective ranges are handled; and
- Whether conclusions are stable across defensible alternative metrics.

The goal is not to find a purely neutral metric; none exists. The goal is to make biological commitments inspectable.

### 6.3 Challenge and recovery paradigms are necessary

If the construct concerns capacity under demand, perturbation-response designs are not optional validation extras. They are closer to the object of interest than resting measurements alone. Repeated within-person measurement is essential because architecture, current state, and context contribute differently to the observed trajectory.

The relevant data are multivariate time series: response onset, cross-system propagation, peak excursion, coordination or decoupling, recovery time, residual effects, and functional continuity. No single challenge is architecture-neutral. Multiple perturbation classes are needed to distinguish a global scalar deficit from an anisotropic robustness-fragility profile. Recent resilience science likewise treats adaptive capacity as something revealed through response to challenge, while network physiology increasingly emphasizes continuous cross-system dynamics rather than isolated physiological snapshots (Colón-Emeric et al., 2025; Munson et al., 2026). The present account extends this direction by making perturbation channel, metric dependence, and viability explicit.

### 6.4 Cost must be measured, not inferred from distance alone

A large biomarker excursion is not necessarily costly, and a modest excursion may require substantial energetic work to contain. Physiological distance from baseline should not be equated with metabolic burden. Where energetic cost is central, calorimetry or metabolic measures can contribute, but one scalar energy measurement cannot identify the full state-specific cost functional without joint trajectory modeling. Functional burden—loss of speech access, mobility, planning, sensory tolerance, or ability to sustain participation—may also be biologically important even when it is not reducible to energy expenditure.

This is particularly important for neurodivergent populations. A behavior that appears externally unchanged may be maintained through high compensatory cost, while an accommodated condition may produce little normative behavioral change but sharply reduce internal burden. Outcome measures that privilege surface normalization can therefore move in the opposite direction from regulatory health.

### 6.5 Relation to data-driven state-space methods

The argument developed here does not privilege hypothesis-specified models over representations learned from data. Contemporary practice increasingly represents physiological and molecular state as a vector and learns its geometry from measurements—through latent-variable models, graph-Laplacian and diffusion-based embeddings, neural differential equations, and hybrid mechanistic–learned formulations—and converges with the present argument insofar as it represents state through structured vectors, graphs, or learned coordinates rather than one scalar summary. The question this section addresses is narrower. Does deriving a representation from measured quantities remove the constitutive choices identified in Section 2 and Section 3, or relocate them?

The choices are relocated, not removed. A graph Laplacian is determined not by the measurements alone but by the feature set, the scaling applied to features, the affinity kernel and its bandwidth, the neighborhood rule, and the normalization adopted. The dependence is explicit and parametric in the diffusion-map construction. Within a one-parameter family of normalizations applied to the same kernel on the same data, α = 0 yields the classical normalized graph Laplacian, α = 1/2 the backward Fokker–Planck operator, and α = 1 the Laplace–Beltrami operator, and the resulting limiting operator and embedding depend on that choice (Coifman et al., 2005; Coifman and Lafon, 2006). These are different limiting operators and embeddings constructed from identical measurements, and the choice among them—typically whether sampling density should influence the recovered structure—is scientifically motivated but not determined by the raw measurements alone. The diffusion-map normalization does not perform the same mathematical operation as selecting the burden metric *W*; both examples show that a representation is not determined by the raw measurements alone.

Comparable dependencies appear in hybrid and physics-informed formulations. Where a mechanistic model is combined with learned components, the strength of the regularization determines how strongly physiologically implausible behavior is penalized and therefore which fitted solutions are preferred (de Rooij et al., 2025). Where a neural network is trained against both data and the residual of a governing equation, the weighting between data-fit and residual terms determines which fitting errors the algorithm penalizes (Raissi et al., 2019). Such weightings are formally analogous to an evaluative cost functional in that they select among candidate representations by penalizing some departures more than others. They are not, without additional biological justification, measures of physiological burden. The analogy is structural: an optimization objective is a declared criterion that shapes what the method recovers, and it should be reported as one. In single-cell and other high-dimensional molecular analyses, normalization, feature selection, embedding dimension, and batch correction can alter the recovered representation and downstream distances, clusters, trajectories, or integration results (Tian et al., 2019). None of this counts against the methods; it means a learned representation is subject to the same requirement as a specified one, and Section 6.2 applies to the procedure that produced it.

Two further requirements from Section 3 apply with particular force to learned representations. First, a learned latent coordinate is a candidate state, and the question of Section 3.1.1—whether it is dynamically sufficient over the modeled horizon—is not settled by predictive accuracy on the training distribution. A representation may predict held-in trajectories well while its future depends on variables it does not encode. Second, the measurement-observability question of Section 3.3.1 does not disappear when the state is inferred rather than specified. When learned coordinates are assigned biological meaning, the relevant issue is whether the state information they represent is recoverable from the measured signals and whether the representation remains stable and transportable. Exact latent coordinates may be identifiable only up to a permutation, rotation, or other admissible transformation, so the requirement is stable equivalence and interpretable observable consequences rather than coordinate uniqueness; for nonlinear models, (*J*, *H*) supplies only the local linear form of the problem. Declaration is necessary but not sufficient (Section 3.1.1): a learned representation must additionally support recoverability to the level the claim requires, stable out-of-sample prediction, and transport across the perturbations and contexts to which the capacity interpretation applies—the requirements of Section 8 and Section 9, applied to the representation rather than assumed for it.

The framework is therefore complementary to data-driven state-space modeling, not an alternative to it: it supplies no better estimator, but specifies what must be declared before any representation, learned or constructed, supports an interpretation as regulatory capacity rather than as a description of a data set under a pipeline. Table 3 sets out where the same declarations reappear.

**TABLE 3:** Constitutive choices under specified and learned representations.

Constitutive choice	Specified state-space model	Learned representation
State space and dynamic sufficiency	Variables selected as regulatory channels; sufficiency over the horizon asserted or tested	Feature set, normalization, embedding dimension, batch handling; sufficiency of learned coordinates must be tested, not inferred from fit
Measurement or encoding map	*H* declared; local reconstruction observability of (*J*, *H*), or the corresponding nonlinear criterion, examined	Measurement channels and encoder or data pipeline declared; recoverability and stability of the represented state information examined, allowing equivalence under admissible transformations
Metric	Burden weight *W*; scaling of state variables	Affinity kernel and bandwidth; neighborhood rule; Laplacian normalization (e.g., the diffusion-map α)
Perturbation ecology	Input channels *B*; perturbation distribution π	Encoded challenge or input variables, sampling design, and the support of conditions and exposures represented in the training distribution
Evaluation or optimization criterion	Biological or functional burden Φ, where claimed	Data-fit loss, regularization strength, physics-residual weighting; not automatically a biological burden measure
Viability	Declared region 𝒱 and endpoint	Supervised outcome labels or thresholds where used; otherwise an external functional or viability endpoint is still required before the representation can be interpreted biologically
Horizon	Declared *T*	Window length, sampling rate, and truncation of the observed series

The rows identify where the same declarations reappear under a different formalism; they are not criticisms of learned representations. A learned representation whose choices are declared and sensitivity-tested satisfies the model-specification requirement of Section 6.2. Interpretation as regulatory capacity additionally requires the identification or recoverability, held-out stability, transport, burden, and viability tests stated above. An unsupervised embedding carries no intrinsic viability meaning.

## 7 Architectural heterogeneity and neurodiversity

The relational formulation provides a mathematical bridge to architectural heterogeneity in biological control systems. Parametric heterogeneity changes values within a shared organization; architectural heterogeneity changes topology, coupling, modularity, timescale organization, or input-output structure. Because robustness is architecture- and perturbation-specific, these differences need not appear as a single severity gradient; they can produce different directions of robustness and fragility across perturbation classes (Csete and Doyle, 2002; Kitano, 2004).

Two cautions are essential. First, a bifurcation caused by changing a parameter is not, by itself, evidence that the underlying architecture changed. A scalar parameter can alter basin geometry or the number of equilibria within one architecture. Architectural claims require evidence about the organization of the control system, not merely nonlinear behavior. Second, one response cannot generally identify architecture separately from state, calibration, and input. Multi-perturbation, longitudinal, and cross-context data are required.

Under the present framework, the empirical signature relevant to architectural heterogeneity is not greater or lesser reactivity, nor failure of a one-scalar rescaling, but a reproducible system-by-perturbation interaction that a sufficiently flexible shared-architecture model cannot explain; flexible parameter changes within a shared topology can alter trajectory shape, timescale, pathway recruitment, bifurcation structure, and viability boundaries. The appropriate null, and the evidence that would support the stronger architectural claim, are specified in Section 8.1 and Table 4 (Prediction 6).

**TABLE 4:** Evidential role, comparator, and disconfirming result for the proposed predictions.

Prediction	Evidential role	Prespecified comparator and expected pattern	Observation that counts against the claim
1. Rankings change across perturbation classes	Core test of scalar versus profile representation	Common-rank scalar model *M*_S_. After conditioning on current state, history, dose, and measurement error, replicated crossing interactions are expected, and *M*_P_ should improve complexity-controlled prediction of held-out responses	The ordering remains sufficiently stable; *M*_S_ is non-inferior or superior; or apparent crossings fail to replicate or disappear after covariates are modeled
2. Metric choice changes classification	Supporting, not specific to RB	Compare a prespecified set of biologically defensible metrics. Changes should be structured and concentrated where profiles are anisotropic or where the metrics price different functions	Substantive conclusions remain stable across the declared metrics, or changes are arbitrary and non-replicable. Metric sensitivity alone does not support RB.
3. Dynamic burden adds information beyond resting measures	Supporting, not specific to RB	Compare resting measures and standard covariates with models that additionally include propagation, accumulated burden, recovery, and residual displacement. Dynamic measures should improve held-out prediction	Dynamic quantities provide no reproducible out-of-sample gain in prediction or mechanistic discrimination
4. Equivalent resting states conceal different response geometries	Supporting evidence for state–dynamics separation	After matching or modeling resting state, exposure history, perturbation dose, and measurement error, systems should retain replicable differences in transient path, burden, recovery, or viability	Trajectories collapse onto a common response family once those factors are accounted for
5. Accommodation effects are channel-specific	Core relational intervention test	Compare a generic-support model with a prespecified profile-by-accommodation interaction. Benefit should be greatest when the intervention reduces demand along the system’s identified fragility direction	No reproducible interaction appears, or targeted and generic supports perform equivalently after dose, expectancy, and baseline state are handled
6. Multi-perturbation data distinguish shared from heterogeneous architecture	Stronger, secondary architectural claim	Compare a flexible shared-topology model with individual-specific parameters against models permitting different topology, coupling, timescale organization, or input–output structure. Architecture-varying models should improve complexity-controlled held-out prediction and remove systematic residuals	The shared-architecture model explains the response family, or added architectural flexibility improves only in-sample fit or produces unstable inferred structures

Predictions 2–4 are expected consequences of the framework but are not specific evidence for Regulatory Bandwidth; they are also compatible with general biological heterogeneity, dynamic-resilience models, and measurement sensitivity. Predictions 1 and 5 are the principal discriminating tests of the conditional-profile account. Prediction 6 concerns a further inference—whether profile differences require heterogeneous architectures rather than different states or parameters within a shared architecture. Simple trajectory nonlinearity is not sufficient evidence of architecture.

This framing is neurodiversity-affirming without insulating the theory from evidence. It does not deny impairment, energetic cost, or episodes of collapse. It changes the explanatory unit. A costly outcome may arise from the interaction between a minority architecture that is coherent under some demand ecologies and an environment concentrated along that architecture’s fragility directions. Support can improve viability by changing the perturbation ecology or distributing regulatory labor, without presuming that health requires convergence toward one normative architecture.

## 8 Falsifiable predictions and a primary model-comparison test

Not every observation compatible with Regulatory Bandwidth is specific to it. Biological heterogeneity alone can produce different responses among individuals; dynamic measurements may improve prediction for reasons unrelated to the present framework; and classifications may change when biomarkers are reweighted. Such findings may provide convergent evidence, but they do not uniquely identify a conditional capacity construct. The primary discriminating claim is narrower: within a prespecified biological domain, a conditional-profile model should predict responses to held-out perturbations or contexts better than an adequately flexible scalar-capacity model. The comparison should hold constant the measurement set, state-variable scaling, primary burden metric, viability endpoint, and time horizon. Metric sensitivity is examined separately; failure of a scalar across arbitrarily changing metrics or endpoints is not evidence for Regulatory Bandwidth.

### 8.1 Scalar-capacity versus conditional-profile models

Let *Y*_ijr_ denote the prespecified scalar response of system *i* to perturbation class *j* on repeated occasion *r*, oriented so that larger values consistently denote greater capacity under the declared criterion; a burden outcome is therefore sign-reversed, whereas viable duration or preserved function retains its natural orientation. Let *s*_ir_ contain measured pre-challenge state, exposure-history, and other occasion-specific covariates.

The scalar-capacity model *M*_S_ assigns each system one latent capacity value *c*_i_:

$$Y_{ijr}=a_{j}+g_{j}\left(c_{i}\right)+\beta_{j}^{T}s_{ir}+\eta_{ijr},$$

where the perturbation-specific intercepts *a*_j_, functions *g*_j_, covariate effects β_j_, and residual η_ijr_ allow perturbations to differ in average difficulty, scale, and state dependence. The residual η_ijr_ absorbs measurement error, unmodeled biological variation, and model misspecification; it is not the measurement error ε of Section 3.1.1. The model is not restricted to equal slopes or linear responses; its defining constraint is that the *g*_j_ are monotone in the same direction across the declared perturbation domain, so one latent value supplies one expected ordering of systems even though their spacing may differ across perturbations.

Because *c*_i_ enters only through *g*_j_ (*c*_i_), its scale is identified only up to a common strictly increasing reparameterization; the hypothesis is therefore ordinal, not metric—after conditioning on the declared covariates, one ordering of systems is preserved across all declared perturbation classes—and a normalization imposed for estimation does not change what is tested. Let μ̃_ij_ denote the adjusted expected response after the declared covariates have been held constant. For any two systems *i* and *k*, and perturbation classes *j* and ℓ, *M*_S_ implies

$$\left({\tilde{\mu }}_{ij}-{\tilde{\mu }}_{kj}\right)\left({\tilde{\mu }}_{il}-{\tilde{\mu }}_{kl}\right)\geq 0.$$

A negative product is a crossing interaction: the expected ordering of the two systems reverses across perturbation classes. Figure 1B provides a deterministic existence proof of such a crossover while holding the burden metric fixed. It is not empirical evidence that the crossover occurs in biological systems. A scalar model could reproduce a crossover by permitting challenge-specific nonmonotone mappings of *c*_i_, but it would then surrender the claim that *c*_i_ defines a stable capacity ordering. Such a model would no longer be the scalar-capacity hypothesis tested here.

Conditioning on measured pre-challenge state is deliberately conservative. Because current state is itself an argument of the Regulatory Bandwidth relation, the adjustment removes part of the phenomenon the profile is meant to represent and raises the evidential bar for *M*_P_; a crossover that persists after adjustment is stronger evidence that the conditional structure is not reducible to measured resting state. It does not isolate architecture and calibration—unmeasured state, incompletely represented history, measurement error, and misspecification may remain—so architectural interpretation still requires the comparison of Prediction 6.

The conditional-profile model *M*_P_ assigns each system a low-dimensional response profile ρ_i_, while each perturbation is represented by a prespecified feature vector ψ_j_:

$$Y_{ijr}=a_{j}+F\left(\rho_{i},\psi_{j}\right)+\beta_{j}^{T}s_{ir}+\eta_{ijr}.$$

The features may include input channel, intensity, duration, predictability, or switching rate; in a simple implementation $F\left(\rho_{i},\psi_{j}\right)=\psi_{j}^{T}\rho_{i}$ , although nonlinear, state-space, graph-based, or hybrid forms may be used. Because different perturbations engage different profile dimensions, *M*_P_ permits reproducible system-by-perturbation interactions and rank reversals. Profile dimensions and perturbation features should be prespecified, independently derived, or regularized strongly enough that the model cannot memorize the training data; prediction to an unseen perturbation class is meaningful only when ψ_j_ is available independently of the outcome, and where perturbations carry only class labels the valid held-out test is prediction of new sessions or contexts within known classes. Because *M*_S_ permits nonlinear monotone *g*_j_ while *M*_P_ in its simple form is linear in the profile, the two models are not nested in general; comparison therefore proceeds by complexity-controlled held-out prediction rather than by a likelihood-ratio test.

Two complementary tests follow. First, the common-rank implication of *M*_S_ can be examined directly through rank-concordance or crossing-interaction tests, with crossings replicated across occasions and estimated with uncertainty; this test is assumption-light with respect to the form of *g*_j_, although it inherits whatever covariate adjustment defines μ̃. Second, *M*_S_ and *M*_P_ are fitted to the same observations with the same covariates and evaluated by a prespecified scoring rule on held-out sessions, contexts, or perturbation classes, with the profile model penalized for its flexibility and the decision rule—including any superiority or non-inferiority margin—declared before fitting.

The conditional-profile formulation gains support within the tested domain when it produces a complexity-controlled improvement in held-out prediction, predicts replicated crossing interactions or channel-specific burden patterns, and yields profile estimates that remain sufficiently stable across occasions. It has no demonstrated incremental value when the scalar model is non-inferior or superior, when apparent crossovers fail to replicate, or when its advantage disappears after current state, exposure history, perturbation intensity, and the participant-, condition-, and session-specific measurement-error contribution have been accounted for. For integrated burden outcomes, that contribution must be estimated and removed, or represented in a joint observation model, as specified in Section 9.1; without that step, a replicated crossing cannot be interpreted as evidence against the common-rank scalar model. Rejection of *M*_S_ supports a conditional representation of capacity. It does not establish architectural heterogeneity. That stronger inference requires a second comparison between a flexible shared-architecture model and alternatives permitting differences in topology, coupling organization, timescale structure, or input–output organization. Simple trajectory nonlinearity is not sufficient evidence of architecture.

## 9 An empirical program

A practical research program can proceed without claiming that the final measure of RB is already known.

Step 1: define the biological question. Mortality risk, energetic burden, functional continuity, recovery time, and viability probability are related but non-identical outcomes.
Step 2: specify the state space and measurement map. Select variables as hypothesized regulatory channels, preserve units until scaling is examined, and check that the measured channels can identify the states over which burden is declared (Section 3.3.1).
Step 3: specify perturbation channels and ecology. Use more than one challenge class where architectural discrimination is an aim; record the frequency and covariance of demands, not only their average magnitude.
Step 4: measure trajectories densely enough to estimate onset, propagation, peak, recovery, and residual state, with contemporaneous functional measures.
Step 5: state and test the metric, making the cost functional explicit and comparing defensible energetic, physiological, functional, and prognostic alternatives.
Step 6: identify viability endpoints, predefining functional destabilization for the studied process rather than substituting normative appearance.
Step 7: report profiles before scalar summaries; any scalar must state its perturbation distribution, metric, horizon, and endpoint.
Step 8: test transportability and competing models across settings, times, and challenge classes, comparing a shared-architecture model with individual parameters against architecture-varying alternatives; architectural interpretations require stronger cross-context evidence than state descriptions.

### 9.1 A worked biological implementation: orthostatic challenge

The eight-step program above can be made concrete through a standardized orthostatic challenge, chosen because the timing and direction of the perturbation can be declared, multivariate signals recorded continuously, and functional stopping criteria specified in advance. This example is a prospective design specification, not a report of results. Head-up tilt and active-standing protocols are established provocative cardiovascular-autonomic tests, and continuous beat-to-beat blood pressure and electrocardiographic heart-rate recording are their standard core measurements (Thijs et al., 2021).

The perturbation may be an active transition from supine rest to standing, a passive head-up tilt, or a set of graded variants differing in transition rate, tilt angle, duration, or concurrent task demand. These protocol properties form the perturbation-feature vector ψ_j_ used by the conditional-profile model of Section 8.1. Let the directly measured physiological signals be represented by

$$y_{ijr}\left(t\right)=Hx_{ijr}\left(t\right)+\varepsilon_{ijr}\left(t\right),$$

where *i* indexes the participant, *j* the challenge condition, and *r* the repeated occasion. Depending on the study question, *y*(*t*) may include beat-to-beat arterial pressure, electrocardiographic beat interval or heart rate, end-tidal carbon dioxide, and cerebral blood-flow velocity or cerebral oxygenation. Symptoms, completion of the standing period, and concurrent cognitive or motor performance are recorded as functional outcomes rather than presumed to be interchangeable with the physiological signals. Lowercase *y*_ijr_(*t*) is the measured time series; uppercase *Y*_ijr_ is the scalar primary outcome constructed from it.

The internal state *x*(*t*) may include hypothesized variables such as vascular resistance, stroke-volume dynamics, baroreflex state, or respiratory–cerebral-perfusion coupling; these are model quantities, and a study that interprets its results as evidence about them, or about coupling or architecture, must examine whether those claims are identifiable from *H*. This distinction matters empirically. In a retrospective head-up-tilt study, orthostatic cerebral blood-flow velocity and end-tidal carbon dioxide predicted lightheadedness, whereas orthostatic heart rate and blood pressure did not (Coelho et al., 2024). That finding does not validate Regulatory Bandwidth. It illustrates why the biological conclusion can depend on which outputs are measured: apparently preserved values in familiar cardiovascular channels do not establish preservation of cerebral perfusion, symptoms, or function across the measured system.

Define departure from the prospectively declared reference trajectory as Δ*y*_ijr_(*t*) = *y*_ijr_(*t*) − *y*_ref,ijr_(*t*). For sampled observations *t*_1_, … , *t*_M_ over horizon *T*, an observed-space quadratic burden can be estimated as

$${\hat{C}}_{Q_{y},T}^{\mathrm{obs}}=\sum_{m:t_{m}\leq T}\Delta y_{\mathrm{ijr}}{\left(t_{m}\right)}^{T}Q_{y}\Delta y_{\mathrm{ijr}}\left(t_{m}\right)w_{m},$$

where *Q*_y_ ⪰ 0 is the declared observed-space burden weight, *w*_m_ is the quadrature weight associated with observation *t*_m_, and *Q*_y_ is not the generic Lyapunov matrix *Q* of Section 3.2. With *L* the observed-space evaluation map of Section 3.1.1, $Q_{y}=L^{T}L$ , and in a noise-free local model this observed burden induces $W_{y}=H^{T}L^{T}LH$ ; if a state-space burden *W*_x_ has been separately declared, the two answer the same question only when *W*_x_ = *W*_y_, and compatibility must be checked rather than presumed whenever interpretation moves between measured-output and latent-state spaces (Section 3.3.1).

The primary *Q*_y_, reference trajectory, and horizon are declared before fitting; sensitivity analyses may compare, for example, a perfusion-weighted criterion with one emphasizing arterial-pressure excursion or chronotropic work, and divergence among defensible criteria may show that systems preserve different functions or incur burden in different channels rather than indicating measurement failure. The primary analysis nevertheless remains fixed so that metric changes cannot manufacture a preferred ordering *post hoc*.

The horizon *T* must be long enough to capture the slow modes relevant to the declared burden, but lengthening it also accumulates measurement error. Under additive, zero-mean measurement error that is independent of the biological signal and has covariance Σ_ε_(*t*_m_),

$$E\left[{\hat{C}}_{Q_{y},T}^{\text{obs}}\right]=C_{Q_{y},T}^{\mathrm{signal}}+\sum_{m:t_{m}\leq T}\mathrm{tr}\left(Q_{y}\Sigma_{\varepsilon }\left(t_{m}\right)\right)w_{m}.$$

For stationary measurement error and regular sampling, the additional term approaches *T* tr (*Q*_y_ Σ_ε_). A longer observation period therefore improves coverage of slowly relaxing dynamics while increasing the accumulated measurement-noise floor. The horizon, sampling rate, filtering, instrument precision, and noise correction must therefore be specified together, and *T* selected relative to the slowest dynamics priced by the burden criterion rather than treating the infinite horizon as biologically natural (Section 3.3.1); process variation ξ(*t*) belongs to the biological dynamics and is not the measurement error ε(*t*) in this expression.

A viability endpoint is defined separately from integrated burden—here, completing the prespecified standing or tilt interval without a safety-mandated termination while maintaining a declared level of cerebral perfusion, symptom tolerance, or task performance. The protocol should terminate at the viability boundary rather than provoke biological collapse; observed termination is therefore an ethically implemented threshold, not evidence of an attractor transition. Recording continues after return to the reference posture so that recovery time and residual displacement can be estimated; return of arterial pressure or heart rate toward baseline establishes recovery only for those variables, not resolution of the broader response, repayment of accumulated burden, or restoration of perfusion, symptoms, and function (Section 4.1).

When the primary outcome is observed quadratic burden, the measurement-error contribution derived above enters *Y*_ijr_ directly. This contribution may vary by participant, challenge condition, session, instrument placement, and signal quality. Because such differences can recur across occasions, a spurious burden difference or crossing interaction may replicate and cannot be excluded by replication alone. Write δε_ijr_(*t*) = ε_ijr_(*t*) − ε_ref,ijr_(*t*) for the measurement error in the reference-subtracted trajectory, which includes error in an estimated reference. Under the stated assumptions, estimate the expected noise contribution as

$${\hat{b}}_{\mathrm{ijr}}=\sum_{m:t_{m}\leq T}\mathrm{tr}\left[Q_{y}{\hat{\Sigma }}_{\delta \varepsilon ,\mathrm{ijr}}\left(t_{m}\right)\right]w_{m},$$

and define the corrected, capacity-oriented burden outcome as

$$Y_{\mathrm{ijr}}=-\left({\hat{C}}_{Q_{y},T,\mathrm{ijr}}^{\mathrm{obs}}-{\hat{b}}_{\mathrm{ijr}}\right).$$

The same correction must be applied before fitting both *M*_S_ and *M*_P_, or represented as a fixed offset within a joint observation model. It should not be included merely as an ordinary covariate with a freely estimated coefficient, because the expected contribution is specified by the measurement-error model, and uncertainty in *b̂*_ijr_ should be propagated into the model comparison rather than treating the correction as known without error. Measurement-error covariance may be estimated from device calibration, redundant channels, signal-quality models, or repeated reference recordings under a prospectively specified procedure; a reference-posture recording alone identifies instrument noise only under additional assumptions, since it also contains genuine biological variation. If the covariance cannot be estimated, or errors are signal-dependent, biased, or correlated with the challenge response, subtracting an additive noise floor is not sufficient, integrated observed burden should not be the primary outcome for the rank comparison, and a viability or recovery-time endpoint—with its own measurement-error, threshold, and censoring model—should be designated primary instead.

The models *M*_S_ and *M*_P_ of Section 8.1 receive the same pre-challenge covariates *s*_ir_, including observed resting state and recorded exposure history. For binary viability outcomes or time-to-event recovery outcomes, the same linear predictor enters through an appropriate generalized-linear link or survival model rather than an additive Gaussian-error formulation. The challenge set should contain enough graded or qualitatively distinct conditions to estimate profile structure—for example active standing and several passive tilt conditions varying in angle or transition rate, with a further condition or repeated session held out—and both models are trained on the same observations, scored against the same primary outcome, and evaluated on the same held-out partition under a prespecified scoring rule and complexity penalty (Section 8.1). Possible primary outcomes include corrected integrated observed burden, probability of reaching the viability endpoint, or time to functional recovery; one should be designated primary rather than allowing the model comparison to select whichever outcome produces the strongest distinction.

The decision rule is that of Section 8.1: the profile account is supported by a reproducible improvement in complexity-controlled held-out prediction, stable profiles across sessions, and replicated participant-by-challenge crossings the common-rank model misses, and has no demonstrated value here if *M*_S_ is non-inferior, crossings fail to replicate, or the advantage disappears once state, history, intensity, and measurement quality are accounted for. Rejection of *M*_S_ would support a conditional representation of capacity, not different regulatory architectures, which still require the separate shared-architecture comparison.

The same trajectories may be analyzed with a specified state-space model, a graph or diffusion-based representation, a learned latent-state model, or a hybrid system; the framework does not decide which will predict best, but requires each to declare what was measured, how the representation was built, what was optimized, and which burden and viability interpretations are assigned (Section 6.5). This example does not report an empirical test of Regulatory Bandwidth. It shows how the framework can be applied to concrete biological observations without conflating measured outputs, constructed burden, latent state, viability, and mechanistic interpretation, and it specifies a comparison in which the scalar model is permitted to win.

## 10 Boundaries and limitations

The formal illustration is local, linear, and time invariant. Whole-organism physiology is nonlinear, stochastic, delayed, switching, and nonstationary. The Gramian formulation does not establish a global theory of regulatory capacity, derive collapse, or prove that a quadratic cost is biologically correct. It shows, under the simplest tractable conditions, why dynamics alone cannot determine burden and why the metric and perturbation distribution are constitutive.

The open-ended character of physiological processes bears directly on the horizon: because regulation in an open system has no natural terminus, no burden or viability claim can be stated without declaring the window over which it is assessed, which is why *T* is an explicit argument of the relation and the infinite-horizon Gramian is treated as a limiting case of the finite-horizon form (Section 3.3.1). That the ordering in the illustration held at every finite horizon examined is a property of the example, not a guarantee: where slow modes are priced, the horizon needed to recover even the ordering may be long. Nothing here supports claims about the long-run outcome of an open system without a declared *T*.

The covariance illustration treats perturbations as random initial displacements. Continuous, colored, or state-dependent forcing requires a driven-system formulation with an explicit input process, the physical input matrix B, and finite-horizon or stationary covariance analysis. The initial-condition result should therefore not be read as a complete model of environmental time series.

Identifying a biologically meaningful cost functional is an inverse problem. Energetic measurements, state trajectories, and functional outcomes may be insufficient to identify all weights uniquely without adequate excitation and modeling assumptions. Different variable selections may produce different apparent modes. These are not small technicalities; they are part of the research program.

Two further identification problems arise once the measurement process is explicit (Section 3.3.1). Whether the state information relevant to a claim can be reconstructed from the instruments is a property of (*J*, *H*); whether an unmeasured direction contributes to the measured and priced output depends on the dynamics propagating it into channels represented by *H* and selected by *L*. Neither can be answered from the channel list alone, and both depend on the coupling structure a study may itself be trying to establish. A separate compatibility question arises when a burden is declared directly on the state, since the state-space and measurement-induced constructions coincide only when $W_{x}=H^{T}L^{T}LH$ ; a study that declares one while computing the other has changed its burden question. These are part of the design specification of Section 9.1 and must be reported.

Where the primary outcome is integrated observed burden, a third constraint follows from the measurement-error contribution derived in Section 9.1: it grows with the declared horizon, can differ by participant, condition, and session, and—because such differences can be stable across occasions—can produce a replicated burden difference or crossing that an uncorrected scalar-versus-profile comparison cannot distinguish from a biological one. It must be estimated by a prospectively specified technical procedure and subtracted as a fixed offset with propagated uncertainty, or represented in a joint observation model; where that cannot be done adequately, an independently defined viability or recovery-time endpoint should be primary, with its own measurement-error, threshold, and censoring model.

Viability boundaries are also scale- and task-dependent. The loss of one function is not equivalent to loss of organismal viability, and clinical thresholds are not automatically dynamical boundaries. Ethical challenge paradigms must avoid provoking collapse in populations already operating near functional limits; Section 9.1 implements this by defining the viability endpoint through prespecified stopping criteria, so that observed termination is an ethically imposed threshold rather than an observed regime transition, and is not evidence of one. Naturalistic and low-intensity perturbations, ecological momentary assessment, and opportunistic longitudinal designs may sometimes be preferable.

The orthostatic implementation of Section 9.1 is a prospective design specification and not a report of results. No data were collected or analyzed for this article, and the specification does not establish that the crossover exhibited formally in Figure 1 occurs in measured physiological systems. What it establishes is narrower: that every declaration the framework requires can be made concretely within an established challenge paradigm, and that the resulting comparison is one in which the scalar model is permitted to win. The empirical test remains to be run; that is the appropriate scope of a Hypothesis and Theory article, and the limitation is stated so that it is not mistaken for a claim the article does not make.

Finally, the argument does not imply that every individual has a distinct architecture or that diagnostic categories map cleanly onto control-system types. Architecture and expressed phenotype need not correspond one-to-one. The claim is conditional: where recurrent differences in coupling, topology, timescale organization, or response geometry cannot be explained by a sufficiently flexible shared-architecture model, architectural heterogeneity becomes the more adequate explanatory unit.

## 11 Discussion

The central result is not that scalar summaries are invalid but that their interpretation is conditional: a recovery rate, resting composite, integrated burden, viable set, or prognostic score may each answer a legitimate question, but none identifies regulatory capacity independently of the system represented, the perturbations considered, the evaluation criterion, and the horizon. Figure 1 makes the issue concrete: two stable local systems with identical spectra and the same asymptotic relaxation rate reverse their apparent burden ranking when either the burden metric or the perturbation channel changes. A scalar may summarize either comparison after its conditions are fixed; it cannot convert the two into one context-free ordering.

A second distinction follows: viability is not adaptation. Remaining within a declared constraint set establishes continued viability for a specified function and horizon, not that the response was beneficial, sustainable, resolved, or adaptive at every level of organization; adaptation is indexed to a biological level, protected function, reference or counterfactual, horizon, and cost criterion (Section 4.1). Regulatory Bandwidth describes conditional perturbation tolerance, burden, recovery, residual state, and viability; it does not assign adaptive value by itself.

This positioning narrows the novelty claim. Regulatory Bandwidth converges substantially with allostasis, dynamic resilience, physiological reserve, robustness, adaptive capacity, and context-dependent stress-response models, and dynamic resilience is its closest neighbor. The incremental contribution is a required conjunction and a construct-identification rule: present regulatory capacity is represented as a conditional profile over a declared system, perturbation ecology, burden criterion, viability endpoint, and horizon before scalarization. Neighboring frameworks can represent the same rank reversals when they retain that structure; the claim is that the structure is the measurement object, not an optional qualification of a person-level score.

The formulation separates observation from mechanism (Section 3.1.1; Section 3.3.1): model state and architecture are model quantities, measured signals are outputs of an observation process, the evaluation map is distinct from the measurement map, and the Gramian represents accumulated burden under a virtual cost output rather than Regulatory Bandwidth, instrument observability, or controllability. Data-driven methods are complementary rather than competing (Section 6.5): they relocate rather than eliminate constitutive choices, and predictive accuracy does not by itself identify a learned coordinate as architecture, adaptation, or Regulatory Bandwidth, just as physiological naming does not validate a mechanistic model. The primary falsifiable claim is comparative (Section 8.1): a common-rank scalar model against a conditional-profile model on held-out sessions, contexts, or perturbations, after current state, exposure history, perturbation intensity, and the participant-, condition-, and session-specific measurement-error contribution have been estimated and removed, or represented in a joint observation model, as specified in Section 9.1. Rejection of the scalar model supports conditionality; it does not establish architectural heterogeneity, which requires a stronger comparison against a flexible shared-architecture model.

For network physiology, this changes how person–environment fit is represented. Capacity is neither wholly inside the organism nor merely imposed by context: architecture shapes how inputs propagate, current state and calibration shape the response available now, the environment determines which perturbations occur, and the evaluation criterion determines which consequences count as burden. Accommodation can therefore have a large biological effect by changing the perturbation ecology or distributing regulatory work without changing the person’s architecture—not an evasion of mechanism but a fuller specification of its arguments. The framing remains neurodiversity-affirming while allowing impairment, high cost, and functional destabilization to be described as outcomes of a brain–body–environment relation rather than as a context-free deficit rank.

Within the Evolutionary Stress Framework, the published Regulatory Bandwidth paper defines the organism-level construct; the present article specifies the measurement architecture needed to operationalize it without false scalarization and provides a comparison in which the scalar model is permitted to win. Local linear tools establish metric, perturbation, and observation dependence under tractable conditions but not global nonlinear transitions, which require separate analyses of attractor change, hysteresis, and collapse, while empirical perturbation studies must determine whether conditional response profiles have stable and useful structure in measured systems. The practical conclusion is direct: perturb carefully, follow trajectories, justify the state and measurement maps, declare burden and viability criteria, model measurement error, and retain conditional response structure long enough to determine whether a scalar summary has earned the claims made for it.

## 12 Conclusion

Biological regulation occurs in networks, under demands, at energetic and functional cost, within boundaries and over time. A scalar measure that omits any of those terms may remain useful for a declared purpose, but it is conditional and partial. Regulatory Bandwidth is therefore best treated not as an amount possessed by a person or as a universal ratio, but as a structured relation among a regulatory system, a perturbation ecology, a burden criterion, a viability endpoint, and a time horizon. The resulting profile describes what the system can tolerate, what the response costs, how recovery unfolds, and where viability is most easily lost; it does not by itself establish that the response is biologically adaptive.

The framework stands or falls through comparative prediction. Within a prespecified domain, an adequately flexible scalar-capacity model must be allowed to compete with the conditional-profile model on held-out sessions, contexts, or perturbations. If one latent value preserves the relevant ordering and predicts the declared outcomes equally well, the profile has no demonstrated incremental value in that domain. If replicated system-by-perturbation crossings and channel-specific accommodation effects are predicted by the profile and missed by the scalar alternative after measurement error and relevant covariates are handled, capacity is better represented as conditional structure than as an intrinsic rank. That result would support a profile, not by itself prove heterogeneous architecture. The stronger architectural claim remains contingent on a separate generative-model comparison.

## Data Availability

The original contributions presented in the study are included in the article; further inquiries can be directed to the corresponding author.

## Author positionality and scope

The author is an autistic scholar and developed the Evolutionary Stress Framework by integrating stress physiology, neurodiversity scholarship, and complex-systems theory. This positionality informed the questions posed but does not constitute evidence for the mathematical or biological claims. The article proposes a theoretical and methodological framework, not a diagnostic instrument, clinical guideline, or individualized medical recommendation.
